# Supplementary material for: A qualitative study of healthy eating, physical activity, and mental health among single mothers in Canada
Source: PLoS One. 2023 Nov 27;18(11):e0294552. doi: 10.1371/journal.pone.0294552 (PMC10681166; doi:10.1371/journal.pone.0294552)
Supplement: S1 Appendix — (DOCX) [file pone.0294552.s001.docx]

**A qualitative study of healthy eating, physical activity, and mental health among single mothers in Canada**

**Appendix 1. Interview Topic Guide**

Interviewer welcomes participants, the aims of the study, and ground rules: There are no right or wrong answers: all of participant’s views are welcome. Audio-recording will start, but if participants do not wish to answer, they can skip that question by simply saying “pass”.

**Introductory questions:**

- Where are you from?
- What language do you speak? If English is not your first language, do you speak fluent English?
- What is your age?
- How many dependent children are living in your home?
- What is your role as a single mother?

**Questions:**

The following question addresses general health about your personal experience on healthy eating, exercise, and mental health. If you feel distressed in the mental health questions, you can skip that question by simply saying “pass”.

- What is your personal experience about healthy eating, exercise, and mental health?

Now we are going to talk about each of those health behaviours:

- With respect to healthy eating, what do you do about maintaining healthy eating?
- What are some of the challenges to maintain healthy eating behaviours?
- What are some of the facilitators to maintain healthy eating behaviours?
- With respect to exercise**,** what do you do about exercising?
- What are some of the challenges to exercise on a regular basis?
- What are some of the facilitators to exercise on a regular basis?
- With respect to mental health, what do you do to balance activities between home and work?
- What are some of the challenges you’ve faced to handle stress?
- What are some of the facilitators to help you move on after a negative event of your life?
- Of all the health promoting behaviours referenced above, what behaviour has been the most important to you in facilitating engagement?

**Prompts:**

- How have you implemented that?
- Was it useful?
- What causes you to feel that way
- Please tell me more…
- Provide examples of these challenges
- Provide examples of these facilitators
- Please elaborate on that
- What’s an example of… (ask participants to expand on an example they had mentioned)
- You said… please expand on …
- How you cope with this
- Talk how is different now than it was. Comparing your life before and after
- What are you going to do in the future related to these practices?
