# Supplementary material for: A qualitative study of healthy eating, physical activity, and mental health among single mothers in Canada
Source: PLoS One. 2023 Nov 27;18(11):e0294552. doi: 10.1371/journal.pone.0294552 (PMC10681166; doi:10.1371/journal.pone.0294552)
Supplement: Attachment — Submitted filename: Response to reviewers.docx [file pone.0294552.s002.docx]

**A qualitative study of healthy eating, physical activity, and mental health among single mothers in Canada**

Manuscript: PONE-D-22-11799

| **Comments** | **Response to reviewers** |
| --- | --- |
| Your justification for the research could be strengthened to really emphasise why this particular research study was needed. | Thank you very much for your comment.  The following statements are added within the last paragraph of the introduction section:  Relatively few articles have captured how single mothers maintain their health, juggle to adapt family responsibilities, and support their children’s health as they follow COVID-19 guidelines.  Qualitative interviews provided in-depth understanding on the challenges and needs of mothers’ health and well-being |
| Could you give more detail on how participants were recruited e.g., how did you contact them? Where did you get their contact details? Etc. Did you aim to recruit participants from different ethnic groups? | Thank you very much for your comment.  More details are provided in the study procedures regarding recruitment of participants:  Participants were recruited through a poster using the official Facebook and Twitter social media platforms of the Hand and Upper Limb Centre (HULC). Then, participants to volunteer for the study contacted the main author by email in which the letter of information and signed consent were provided through a Western Qualtrics online form prior to the one-on-one interviews.  More details are provided in the results regarding ethnicity:  Out of the twelve single mothers, two identify their ethnicity as Korean, two as Indian, as the remainder as Canadian. |
| In table 1 the ‘city’ column seems to indicate some mothers were from the UK but the title state ‘Canadian single mothers’- please clarify. A separate section on ‘Analysis’ is needed and more detail on this is needed. You state the analysis is ‘theory-driven’ but do not say how or which theory/ theories are being used. You state the authors ‘become co-participants’ but more detail is needed on what exactly this refers to. Did you use NVivo or any other software to manage the analysis? Please provide a reference for ethical approval. | Thank you very much for your comments.  Please, see below the improvements:   - As London is a city of Canada, we have included the province of Ontario. For example, London, ON. - In terms of theory-driven, we removed that terminology as we are not using a particular theory. - Co-participants acknowledge the co-construction of the research-participants relationship and the importance of engaging participants in research process. - We used NVivo 12 software to facilitate coding and analysis of participants’ responses. - The reference number for ethical approval is provided as well as the date of approval |
| The results are an interesting read, and the quotes really illustrate the hardships the participants are facing. Overall, the results are too long and could be more succinct and less repetitive in places. The subtitles are too long and should be more succinct. It would be helpful if the subtitles were the same as the 3 themes in table 2. Be careful not to overgeneralise your results eg. rather than reporting your findings as ‘Canadian single mothers…’ or ‘Single mothers’, say ‘Participants….’ | Thank you very much for your comments.  Please, see below the improvements:   - To make the results more succinct, we subtracted the whole section under Table 2. This allows the reader to directly go to theme 1 of table 2. - We changed single mothers in Canada as well as participants instead of Canadian single mothers. These changes were applied for title and content. |
| The discussion repeats some of what is written in the results but needs to focus more on how the findings relate to the other literature. Your discussion should have a section on the strengths and limitations of the study (Eg. Sample, methods, data quality eg did online interviews impact the data quality?) | Thank you very much for your comment.  Strengths and limitations is a new section added into the manuscript. |
| I think the recommendation of providing food kits is good. Is there anything specific that could be recommended in relation to physical activity? | Thank you very much for your comment.  We wrote the following implication:  It is recommended to plan physical activity on moderate intensity levels and regular leisure time routines. Single mothers will be more likely to plan physical activity if they feel more confident in their ability to engage in physical activity and would lessen the stressors of home and family responsibilities. |
| I think most of the findings apply to non-single mothers stuck in quarantine situations at home with their kids. For example, things like “going back to the basics (line 334)” in reference to starting outside walks are applicable to a wide demographics of people who are stuck at home under pandemic closures. Same is true with exercising using online videos etc. Small sample size of 12 is not helping here either as it is not convincing. | Thank you very much for your comment.  These are reflected in a new paragraph called Strengths and Limitations. |
| Being a Canadian vs American or for that matter from any other developed country doesn’t seem to matter in the content of the survey performed. If fast food is easily available and fruits and vegetables could be purchased, should one want to engage in healthy eating, (which is typical for any developed country), the findings could be extrapolated to other developed countries (at least in recommendations/future research section). | Thank you very much for your comment.  This comment was incorporated as a limitation and statement of a future research endeavour at the end of the implication sections. |
| I suggest authors should think about generalizing the outcomes of the study to generate a novel enough content to warrant publication. My suggestion is to cut down on quotes from the respondent’s surveys as those are too lengthy for the value they contribute. I do not find much value in those quotes. Instead, I would like to see literature on behavioral outcomes including for example eating habits resulting from stress (as it could be applicable here), including during prolong conflicts such as war. I suggest adding research limitation section where the small sample size should be addressed. | Thank you very much for your comment.  We cut off additional information, specifically the whole section under table 2. Additionally, a limitation section was included in which we mentioned we had a small sample size. |
| Additional grammar errors to correct Line 111 delete “of resilience” Line 144 change transfer to transferred Line 247 change access to accessible Line 263 change thy to they | Thank you very much for your comment.  Suggestions are applied. |
