# Supplementary material for: A qualitative study of healthy eating, physical activity, and mental health among single mothers in Canada
Source: PLoS One. 2023 Nov 27;18(11):e0294552. doi: 10.1371/journal.pone.0294552 (PMC10681166; doi:10.1371/journal.pone.0294552)
Supplement: Attachment — Submitted filename: Response to Reviewers.docx [file pone.0294552.s003.docx]

**A qualitative study of healthy eating, physical activity, and mental health among single mothers in Canada**

Manuscript: PONE-D-22-11799

| **Comments** | **Response to reviewers** |
| --- | --- |
| This is an improved version of the paper and I appreciate your efforts in trying to incorporate the reviewer’s feedback. I still find issues with the way paper is presented and I summarized most of them below. 1) I still find that the quantity of quotes offered in sections following table 2 are excessive for the value they bring to the paper. I do not find them to be insightful. I would like to see fewer quotes. I would rather see themes and percentages of respondents who mentioned a particular topic and/or responded to a particular theme, than a quote from one person. Alternative way to handle quotes could be creating an appendix and putting all quotes into the Appendix and just referring the reader to the Appendix if the reader wants to see a particular quote. | Thank you very much for your comment.  The quantity of quotes has been minimized following table 2. |
| 2) Abstract: Lines 24 and 33: you refer to “dual parent families” in line 24 but you also refer to “parents” in line 33 as grandparents of a child. Please sort out this confusion as it is not clear in the abstract before the reader knows anything about your study whether you are referring to single parent or multi-parent household. I would suggest calling mother’s parents everywhere in the paper as grandparents. It will avoid any confusion. | Thank you very much for your comment.  The change of terminology was addressed. |
| 3) Line 62: I do not see that it was illustrated before that single mothers are employed in low-paying jobs. This is taken out of context. Please provide references or create context so that it is clear that you are looking at the low-income community, for instance. | Thank you very much for your comment.  Line 62 was removed as this is out of context |
| 4) Line 187: replace “as the remainder” with “and the remainder.” | Thank you very much for your comment.  Correction was made |
| 5) Table 1: why are some responsibilities stated as providing emotional support to a child and some are not? What does it mean when a mother does not have this responsibility? Does it have a significant impact on the outcomes? Also, some responsibilities are listed as shared with parents. Whose parents? Mother’s? This is a perfect example of the continued confusion of using the same “parent” term for mother and grandparents. Please consider changing to grandparents. Please clarify all these nuances, as it is not clear if these differences make a significant impact on the results. If these are not relevant, then please use the same language to describe duties of each mother. If these are significant differences, then please offer some discussion. | Thank you very much for your comment.  Emotional support to the child was removed as this concept is not in the discussion section. |
| 6) Lines 214-215: Sentence “However, …” Please rewrite this sentence to indicate that single mothers were not eating junk foods because they were aware of negative health impacts of such foods. | Thank you very much for your comment.  Sentence was improved. |
| 7) Lines 500 – 503: You need to expand your process of thinking here because it is not clear what you mean. Why is the fact that findings can be generalized to non-single mothers is a limitation? Shouldn’t it be a strength? Please elaborate on every strength and every limitation.  This entire added section “Strength and Limitations” needs to be re-written as it reads as perfunctory text with not meaning. It is not enough to acknowledge a limitation/shortcoming by stating it’s existence You also need to offer a strategy of how to deal with it in another study, or in a future research. For example, why is your sample so small? What can you do differently in the design of another study to generate a larger sample? Perhaps you can say that next study should include all mothers, not only singles. You need to offer practical strategies for tackling listed limitations. | Thank you very much for your comment.  The suggested edits were applied within limitations. |
