# Supplementary material for: A qualitative study of healthy eating, physical activity, and mental health among single mothers in Canada
Source: PLoS One. 2023 Nov 27;18(11):e0294552. doi: 10.1371/journal.pone.0294552 (PMC10681166; doi:10.1371/journal.pone.0294552)
Supplement: Attachment — Submitted filename: Response to Reviewers.docx [file pone.0294552.s004.docx]

**A qualitative study of healthy eating, physical activity, and mental health among single mothers in Canada**

Manuscript: PONE-D-22-11799

| **Comments** | **Response to reviewers** |
| --- | --- |
| This is a much-improved paper. It reads well. My only suggestion is to rework the Strengths and Limitations section as it is still disjoint and weak compared to the rest of the paper. I offered my additional suggestion below. | Thank you very much for your comment  See the comments at the end of this document |
| Page 7; Section Materials and Methods  Complete the following sentence: “First, specify an initial sample size upon which is the first round of analysis.” | Thank you very much for your comment.  Sentence is now complete |
| Table 1:  For entry 10 is stated as preparing meals while others do not have this as listed responsibility. Please either remove this or add to other mothers for consistency in describing their responsibilities. | Thank you very much for your comment.  This was removed |
| Page 18; Section Barriers of mental health  Delete “were exacerbated” from the following sentence: “Additionally, limited contact with their loved ones triggered isolation, which contributed to exacerbation of their depressive symptoms were exacerbated.” | Thank you very much for your comment.  This was deleted |
| Page 23: Section Strengths and Limitations  This is the weakest section and needs additional work as it reads disjointly.  I suggest perhaps changing this section to Limitation only.  Within the text, you can then say that while your study successfully addressed diversity, etc., it also may have suffered from cultural bias, small sample effects, etc….  The following sentence should be modified:  “Most of the findings applied to mothers in general such as being stuck at home with children, outside walks, online video exercising.” What are you trying to say here? Many of the finding from this study are applicable to a wide pool of mothers? Or that the findings from this study are generalizable to a wider selection of mothers? Please clarify. If that is the case, then you can say that future research may want to extend/repeat similar study but include a wider pool of mothers. However, Covid pandemic is over and the opportunity to collect similar data is over. Thus, perhaps you can suggest a different context when mothers are stuck at home (unemployed, poor, etc. ).  Statements relating to small sample size do not make sense in the context of repeating the same study for all mothers:  “Finally, the sample size was small. To tackle all of these limitations, the next study should include all mothers, not only single mothers.”  Pandemic is over, thus recreating the same conditions will not be feasible. You need to come up with similar circumstances to suggest recreating a similar study. | Thank you very much for your comment.  Suggestions are implemented throughout the paragraph |
